# ‘How shall we survive’: a qualitative study of women’s experiences following denial of menstrual regulation (MR) services in Bangladesh

**DOI:** 10.1186/s12978-016-0199-8

**Published:** 2016-07-22

**Authors:** Altaf Hossain, Heidi Moseson, Sarah Raifman, Caitlin Gerdts, Kamal Kanti Biswas, Diana Greene Foster

**Affiliations:** Association for Prevention of Septic Abortion, Bangladesh (BAPSA), Dhaka, Bangladesh; Department of Epidemiology and Biostatistics, University of California, San Francisco, CA USA; Advancing New Standards in Reproductive Health (ANSIRH), University of California, San Francisco, Oakland, CA USA; Ibis Reproductive Health, Oakland, CA USA; Ipas Bangladesh, Dhaka, Bangladesh

**Keywords:** Menstrual regulation, Abortion, Denial of abortion, Second trimester, Bangladesh

## Abstract

**Background:**

About one quarter of women in Bangladesh are denied menstrual regulation (MR) due to advanced gestation [J Fam Plann Reprod Health Care 41(3):161-163, 2015, Issues Brief (Alan Guttmacher Inst) (3):1-8, 2012]. Little is known about barriers to MR services, and whether women denied MR seek abortion elsewhere, self-induce, or continue the pregnancy.

**Methods:**

After obtaining authorization from four health facilities in Bangladesh, we recruited eligible and interested women in to the study and requested informed consent for study participation. We conducted in-depth interviews with 20 women denied MR from four facilities in four districts in Bangladesh. Interviews were translated and transcribed, and the transcripts were analyzed by two researchers through an iterative process using a qualitative content analysis approach.

**Results:**

Of those interviewed, 12 women sought abortion elsewhere and eight of these women were successful; four women who sought subsequent services were denied again. Two of the eight women who subsequently terminated their pregnancies suffered from complications. None of the participants were aware of the legal gestational limit for government-approved MR services. Given that all participants were initially denied services because they were beyond the legal gestational limit for MR and there were no reported risks to any of the mothers’ health, we presume that the eight terminations performed subsequently were done illegally.

**Conclusions:**

Barriers to seeking safe MR services need to be addressed to reduce utilization of potentially unsafe alternative abortion services and to improve women’s health and well being in Bangladesh. Findings from this study indicate a need to raise awareness about legal MR services; provide information to women on where, how and when they can access these services; train more MR providers; improve the quality and safety of second trimester services; and strengthen campaigns to educate women about contraception and pregnancy risk throughout the reproductive lifespan to prevent unintended pregnancies.

**Electronic supplementary material:**

The online version of this article (doi:10.1186/s12978-016-0199-8) contains supplementary material, which is available to authorized users.

## Background

Unsafe abortion is one of the leading causes of maternal mortality [1]. The majority of all abortions in developing countries are performed in unsafe or illegal conditions and the consequences can be severe [2]. This is despite the fact that in the vast majority of countries abortion is legal for one or more indications [3]. Factors such as poverty, stigma, lack of awareness of the law and distance from a provider prevent women from accessing safe abortion services within government authorization [4, 5]. As misoprostol use becomes more common in settings where abortion is restricted, it is important to identify predictors of seeking informal sector abortion and its health consequences.

Abortion is illegal in Bangladesh except to save the woman’s life [6, 7]. Menstrual regulation (MR) services, however, have been authorized by the Bangladesh government since 1979 [8]. MR commonly consists of manual vacuum aspiration (MVA) to safely establish ‘non-pregnancy’ after a missed period [9]. Since mifepristone was approved in Bangladesh in 2013, it is also used for MR, in combination with misoprostol [10]. Because pregnancy is not clinically diagnosed prior to the procedure, MR is considered a backup family planning method rather than an abortifacient. The government approves physicians and trained paramedics to provide MR up to 10 and 8 weeks post last menstrual period (LMP), respectively [8, 11, 12]. As of 2011, the total number of health professionals trained in MR included 10,600 doctors and 7,200 paramedics, primarily female family welfare volunteers (FWVs) [13]. Many attribute Bangladesh’s significant reduction of maternal mortality in recent decades [14] to sustained declines in abortion-related deaths and increased availability of MR [13, 15, 16].

Though government approved and free of cost, MR services are still difficult to access for many women in Bangladesh [17, 18]. Recent evidence shows that approximately one quarter of women are denied MR services annually [13, 19]. Another study shows that more than three in ten facilities reject women’s requests for MR for reasons that are not sanctioned by the government, including being young, single, nulliparous, or lacking husband’s consent [5]. In 2010, only 57 % of designated facilities actually provided MR services, due to insufficient equipment, shortages of trained staff or both [5]. Additionally, low knowledge of MR among women further limits access [20].

As a result of limited access to government approved services, a significant number of women seek care outside of the formal health sector, which is more likely to be unsafe. In Bangladesh, approximately two pregnancies are terminated for every five live births. Half of these terminations (equivalent to about 653,000) result from government sanctioned MR procedures, performed by a trained provider in a facility and within the permissible number of weeks post LMP, and half (equivalent to about 647,000) result from induced abortions, defined as the termination of a pregnancy by a procedure or action taken by a provider or a woman herself, outside the definition of MR [5]. Complications from MR remain high—approximately 120 out of every 1,000 procedures [5, 12], which is higher than would be expected based on the safety of MVA in other settings [21]. Induced abortions have a complication rate three times higher than that of sanctioned MR procedures—358 out of every 1,000 illegal abortions [13].

Although it has been documented that women are denied government approved MR services, no evidence exists on what happens to women after denial [13]. As part of a larger multi-country Global Turnaway Study, this study aims to understand the experiences of women denied MR in Bangladesh. Similar studies have been completed in South Africa, Nepal, Tunisia and Colombia [22–24]. Specifically, we investigate women’s pregnancy decision making process, reasons for their denial of MR, the barriers they confront in obtaining MR, and where they go after denial of MR.

## Methods

Women were recruited using purposive sampling from a total of four facilities in Rajshahi (10), Dhaka (2), Manikganj (4), and Narayanganj (5) districts. The purposive sampling strategy aimed to obtain a diverse range of experiences from both public and private facilities in both urban and rural areas. Specific clinics were identified with assistance from local partners working within Bangladesh at a safe abortion organization, Association for Prevention of Septic Abortion, Bangladesh (BAPSA). The four selected facilities included two public facilities (9) and two private non-governmental organization (NGO) facilities (12). The selected public facilities represent different levels of the public health system: a mid-level public Maternal and Child Welfare Center and the Upazila Health Complex to represent the rural centers and lower-level facilities. We did not select a facility within the lowest administrative unit, Unions, due to the low caseload in these areas; however, the Upazila Health Complex serves as a central point of care for many rural women, who live in the villages surrounding it. The two private NGO facilities were selected because they are well-known to be large providers of abortion care in the country.

Recruiters conducted eligibility screenings in February 2014 with women presenting for MR at each recruitment facility and obtained informed consent and contact information from all those interested in participating in semi-structured qualitative interviews. Recruiters explained to women that the interview questions would cover their experiences seeking MR, knowledge about the abortion and MR laws and services, reasons for being denied MR and experiences with MR services generally. There was no explicit explanation that the study would focus on the participants’ “next steps” so as not to influence the behavior of study subjects. Women were eligible for the study if they were 18–49 years old and denied MR services on the day they were recruited. In-depth interviews were conducted two months following recruitment, in order to allow time for women to pursue a course of action following denial. Interviews were conducted face-to-face in Bengali and audio-recorded at the participant’s residence, or preferred location, and lasted a duration of one hour. Researchers obtained verbal consent at the time of recruitment and again at the time of interview, in addition to audio-recording consent. Interviewers were trained in qualitative research methods and used structured interview guides with prompts to ask women about their MR decision making process, their reactions and actions after denial of services, and knowledge about MR and self-induction [Additional file 1]. In addition, women were asked questions pertaining to their socio-demographic and economic background. A professional translator was hired to translate the transcripts from Bengali to English for analysis.

Transcribed data were analyzed using a qualitative content analysis approach, using a consistent set of codes to organize text with similar content after data collection was completed, transcribed and translated. Two researchers, trained in qualitative methods, separately performed open coding on the same two transcripts, and then together developed a preliminary codebook. The codes generated were influenced by other studies previously completed as part of the same global study on denial of abortion, in Tunisia, South Africa and Nepal [22–24]. The researchers used this codebook to separately code five additional transcripts and then further refined the codebook as needed. The two researchers coded the remaining transcripts using the revised codebook and, together with the larger research team, interpreted the data and identified key quotations to include in this manuscript.

The Bangladesh Medical Research Council (BMRC/NREC/2010-2013/1310) and the University of California, San Francisco Committee on Human Research (IRB#10-045110) granted ethical approval. Quotes are non-identifiably attributed to respondents using the first letter of the district in which the interview took place and a number, indicating the order of the interview. The pregnancy outcome (induced abortion, spontaneous abortion, or carried to term) is also included in parentheses after each participant quotation. We use the broader term “abortion” for the pregnancy outcome and in reference to women who attempted to terminate a pregnancy following the denial of MR because it is unclear whether or not the services rendered in all cases were government sanctioned MR; in many cases the window for government sanctioned MR had already passed.

## Results

A total of 44 participants were recruited. Researchers were able to follow up with 33 study participants two months later. Of these 33 women, 21 agreed to be interviewed. The remaining 12 no longer wished to participate in the study. Eleven women were lost to follow up because interviewers were unable to contact them despite several attempts to reach them using the contact information they provided at recruitment. One woman was ineligible due to the fact that she was 14 years old. After 20 interviews, data collection was deemed complete due to limitations of project funding and staff availability. However, key themes were repeated across many of the interviews, which provided encouraging evidence that, if not fully achieved, collected data were near saturation.

The mean age of the study participants was 27 years old (SD: 6.6, range: 18 to 39). All of the participants were married and the mean age at marriage was 16 years (SD: 2.6, range: 12–21). Five participants did not have any children, while the rest had between one and four children (Table 1). The average monthly income was about 8,500 taka or about 108 US dollars (ranging from 2,500 to 15,000 taka), with eight participants at 10,000 or more taka, 6 between 5000 and 10,000 taka, and 6 at 5000 taka or less. Most participants (14) had secondary or higher education, a much higher proportion compared to national averages which report 54 % of the secondary school age female population attended secondary school in 2014 [25]. Most participants (16) reported their occupation as “housewife”, two reported that they were students and two did not indicate an occupation. Fifteen participants reported that they were living with their husbands at the time of the interview, four did not indicate either way, and one said she was living with her parents because she did not have a good relationship with her husband.

**Table 1:** Demographic characteristics & care-seeking experiences of sample

Means	Mean (SD)
Age, years	27 (7)
Age at Marriage, years	16 (3)
Number of Children	1.6 (1.2)
Daughters	1 (1.1)
Sons	0.7 (0.7)
Frequencies	N (%)
Education	
Missing	1 (5)
None	2 (10)
Primary	3 (15)
Secondary	11 (55)
College	3 (15)
Spouse’s Education	
Missing	1 (5)
None	4 (20)
Primary	9 (45)
Secondary	4 (20)
College	2 (10)
Average Monthly Income, taka	
5,000 or less	6 (30)
Between 5,000 and 10,000	6 (30)
10,000 or more	8 (40)
Main Occupation of Respondent	
Housewife	18 (90)
Student	2 (10)
Place of Living	
City/Town	5 (25)
Village	15 (75)
Cohabitation with Man involved in the Pregnancy	17 (85)
Contraceptive Use	
None	11 (55)
Condoms	5 (25)
Oral Contraceptive Pills	2 (10)
Injectables	1 (5)
Pregnancy Unintended	18 (90)
Reasons for MR^a^	
Care for other children	7 (35)
Limited resources	6 (30)
Sex of child	2 (10)
Maternal Age - too young	1 (5)
Maternal Age - too old	4 (20)
Fear fetal malformation	1 (5)
Marital Issues	2 (10)
Reasons for Denial of MR	
Gestational age beyond limit	20 (100)
Maternal health concerns	9 (45)
Sought abortion elsewhere	12 (60)
Illegal abortion successful	8 (67)^b^
Complications of illegal abortion	2 (25)^c^

^a^More than one reason per respondent is recorded, hence, frequencies and percents sum to more than the total

^b^This percent reflects percentage among women who sought an illegal abortion

^c^This percent reflects the percentage among women who succeeded in getting an illegal abortion

All of the study participants were told that they were beyond the gestational limit for MR services, most often indicated as 12 weeks gestation. Ultimately, 11 participants carried their pregnancies to term, eight had induced abortions and one had a spontaneous (though wanted) abortion. Twelve out of the 20 participants sought services at more than one facility, including all of the participants who terminated their pregnancies (8) and four of the participants who ultimately carried to term. Of those four who sought services elsewhere but ultimately carried to term, three were denied again due to gestational age and one participant decided against it after learning about the risks of second trimester abortion. Of the eight participants who obtained induced abortion, two had complications, including severe bleeding, one of which required surgery (Table 1).

Eight participants were using contraception when they became pregnant (Table 1). Many of the remaining participants (12) believed they were at low risk of becoming pregnant for various reasons, such as delayed return to menstruation after childbirth, recent removal of contraceptive implant, older age, previous abortion(s), or severe menstrual pain. The return of menses following childbirth or breastfeeding can vary from woman to woman, and many participants did not know that they could get pregnant in this window: ‘My youngest child is two years old and I didn’t have menstruation since [he was born]. I had heard from others that after having a baby you cannot get pregnant unless your menstruation is back. So I didn’t take any contraceptive’ (M2, carried to term).

### Pregnancy recognition

Some participants, both those who ultimately had terminations and those who carried to term, reported that they did not recognize the pregnancy until they were beyond the gestational limit for MR, at which point they could no longer qualify for MR services during the government approved gestational window. Many participants were delayed recognizing their pregnancy due to misconceptions about pregnancy risk or irregular menstrual cycles. One participant described: ‘I couldn’t identify the pregnancy early as my period was very irregular and takes place in three to four month gaps. When the doctor told me about the pregnancy, it was already more than three months’ (R3, induced abortion). Other participants were either not familiar with pregnancy symptoms, had attributed pregnancy symptoms to ill health, or had been using contraception and did not expect that they were at all susceptible to becoming pregnant.

### Reasons for seeking MR

There was little difference between the reasons for seeking MR between those participants who ultimately obtained the services and those who did not. Concerns about providing care for multiple children, financial stress due to poverty, or being too old or young were the most frequently cited reasons for abortion-seeking. Seven participants explained that they wanted MR because their young children still required their attention and resources. For example, one participant said: ‘I have a child who is two years old and I have to spend all my time to look after the baby. In this situation taking another baby was not wise and I felt it was an extra pressure. So I thought of getting an abortion’ (D1, carried to term). Four participants mentioned limited financial means as their primary reason for seeking MR. One explained: ‘If we keep this pregnancy, I have to leave my job. In this circumstance we cannot afford to have a child. That was the reason we decided to terminate this pregnancy’ (S3, carried to term). Another participant said: ‘I felt bad. I have three kids and all of them are very young. How can a poor family like ours raise so many kids? My husband also got worried. We are a very small farmer family. How shall we survive with four kids?’ (R5, induced abortion).

Women’s age was also a factor in deciding to pursue MR services. A few participants, who had no children, felt that they were unprepared to start a family, while several others said they were too old to have another child and that their existing children were already grown up. One participant said: ‘At the age of 39 when my youngest child is 15 years old and proposals are coming for my elder daughter, it did not seem to be the right thing to have a child at this time. […] This will be socially very embarrassing if we have a child at the moment. People will laugh at us’ (M3, induced abortion).

Two participants reported that the sex of the fetus was the primary reason for seeking MR. Both were in their late-30s, had nearly grown daughters (two and four daughters respectively), and were expecting another girl child. One of the participants was unable to confirm the sex of the fetus until her seventh ultrasound at seven months gestation, at which point she was denied MR (M1, carried to term). She said: ‘My future plan is to raise these five kids and take care to avoid unwanted pregnancy. I shall always take family planning methods from now on’. The other participant reported that she obtained an abortion at a private clinic after being denied MR due to gestational age because she ‘was sure that this pregnancy is also a girl (I had the intuition that I shall never have a son)…’ (M3, induced abortion).

### Reactions to denial of services

All the participants in this study were denied MR services due to gestational age. Most reported a gestational age of three to four months at the time of service denial. Participants commonly reported that providers told them MR could be dangerous for their health so late in the pregnancy. As a result, after being denied services, nearly half of the participants (8) resigned to continue their pregnancies and not seek MR services elsewhere, particularly after talking with their husbands. One participant said: ‘I told my husband that the doctor has told me that MR cannot be done at this stage. That’s what he wanted. He advised me to continue the pregnancy. My mother also wanted me to do the same’ (R7, carried to term). Another participant seemed to take the denial at face value:

*‘After I returned from the clinic, I did not talk to anybody and also did not take any advice from anybody. Only informed my parents that this pregnancy cannot be terminated. I did not think of visiting another clinic… I have heard from the neighbors that termination of longer duration pregnancy can cause damage to a mother’s health and the mother can even die. I have decided to continue the pregnancy’ (M4, carried to term).*

Some of the participants who carried to term accepted the reality of the situation and appeared to be positive about their future. One said: ‘There is no problem with this pregnancy in my family. Primarily we wanted to abort this pregnancy as we have a young kid. There is no problem now. My husband just wants a healthy baby. I expect this baby will have a good influence in our life. We’ll have two kids and will try to raise them with good manner’ (R6, carried to term). Others were a little less optimistic; one participant explained: ‘For the first few days I treated my husband badly. I was upset as my schooling has been interrupted at least for 1 year. Now it is OK. I think it will not affect anything of my life. Only my schooling has stopped for 1 year‘(R2, carried to term).

The remaining 12 participants, all denied MR services due to gestational age limits, sought abortion services elsewhere. One participant explained her reaction after being denied: ‘What shall I do now? I don’t want to have any more children. My sister told me that she knows a village doctor who performs MR by medicine’ (R10, induced abortion). Eight of the participants who sought services elsewhere ultimately obtained an abortion and four were again denied due to gestational age. One reported that she had a spontaneous abortion the day after being denied services from a clinic (R4, spontaneous abortion). One participant explained: ‘After returning from the hospital I talked to my husband and also to my mother and they advised me to go to a private clinic and listen to what they say’ (R3, induced abortion). Another said: ‘As I did not want to keep this pregnancy, I discussed with my mother and my sister at my mother’s home about where to go next for safe MR’ (R9, induced abortion).

### Support and advice from others

Participants chose to disclose their pregnancy and their decision-making process around termination with a variety of individuals, at different times. Specifically, many sought support and advice when (1) confirming the pregnancy, (2) deciding whether and how to seek MR, and (3) what to do following the first denial of MR services. Whether and who they consulted, however, varied across these three time points.

Some participants spoke only with their husbands at all three decision points. One explained: ‘I did not discuss the pregnancy with anyone other than my husband. My husband advised me not to disclose this information to any third person and told me that we cannot have a child in this situation’ (S2, induced abortion). For those who told their husbands, many of the men were unhappy or concerned. One participant recounted that her husband was upset and blamed her: ‘When my husband found out about the pregnancy he became angry and accused me of taking the child deliberately’ (D1, carried to term). Some participants delayed seeking MR because they needed time to persuade their husbands or families, and to plan and prepare for the clinic visit. One explained: ‘There was no difficulty making the decision. Only it took some time to convince my husband. It took 4 days to go to the clinic after I decided to go for MR’ (M2, carried to term). Three participants’ husbands were indifferent to the pregnancy. And others said their husbands were happy about the pregnancy, even when the participant herself did not want to carry to term (S3, carried to term; R7, carried to term).

Most participants, however, discussed the decision-making process with a friend, family member, or neighbor, in addition to their husbands. The majority of participants sought the counsel of these individuals, but still felt that they made the final decision on their own (11), or together with their husbands only (8). The influence of family and friends was strong in some instances. Several participants reported that family members persuaded them to seek abortion even when their initial instinct was to continue the pregnancy: ‘I did not have a good relationship with my husband and was living with my parents. I wanted to keep the pregnancy. But, my mother was totally against it. She was in favor of terminating the pregnancy. I had no other choice than to follow her orders’ (M4, carried to term). Conversely, some participants received advice to continue the pregnancy, yet were able to convince family members that seeking MR was the right decision for them: ‘[My landlady] suggested that I keep the pregnancy, as it was my first conception. I told her that I can’t continue the pregnancy and explained the situation and hardship, and that in this situation I have no alternative but to go for MR. Listening to this she told me about the hospital. She also helped me to go there’ (S3, carried to term).

After participants were denied MR services the first time, however, they often lacked sufficient resolve to stand up to familial pressure a second time. The participant whose landlady and husband were initially against the abortion apparently gave up on MR services after having been denied MR at the hospital. She explained:

*I have discussed with my husband and my landlady after returning from the hospital. My husband tried to convince me and advised me to keep the pregnancy and so did the landlady. I thought that someday we have to have children, and it was our first child. Moreover, doing MR is risky at this stage. I have changed my mind and was convinced to continue the pregnancy (S3, carried to term).*

Another participant in a similar situation said:

*I talked to my husband, aunty and my younger sister. Primarily they advised me not to go for MR. But I was firm in my decision and they had to support me at last. My younger sister took me to the private clinic and later I went to government maternity with aunty. My mother was also informed […] After returning from maternity I discussed with my husband and my mother. Both my mother and husband advised me to give up the abortion idea. There is no difference having four or five children. My mother said, “If you are unable, I shall raise your kid.” (M1, carried to term)*

Almost all of the participants who were ultimately successful in obtaining an abortion learned about alternative providers from family members or neighbors.

### Delays and barriers in seeking MR

In addition to late pregnancy recognition, participants were delayed for other reasons including time needed to make the decision; changing relationship dynamics with a partner; and logistical concerns relating to employment, childcare, locating a provider, securing funds, and more. One participant’s response highlights the interplay of factors leading to her delay, from lack of information on providers, to conflicting feelings about the pregnancy, to fears of stigma and mistreatment, to concerns about financial cost:

*There were some difficulties I faced in making the decision. First at the pharmacy, they told me that if the medicine fails to terminate the pregnancy then I have to go to hospital for MR. I thought that if I have to go to hospital for MR after the failure of medicine, then it will be a physical harassment for me and also financial loss. I was in a dilemma and it took some time to make a decision. It took a week to go to the provider after making a decision. I was afraid of uncertainty resulting into late decision making. Also I didn’t know where to go for MR. (D1, carried to term)*

Family illness or employment obligations also delayed the MR-seeking process for some participants. Due to obligations such as caring for children and other family members or due to inability to secure time off from employment, several participants reported delays of more than one month.

Delays seeking services may have led to participants being denied government approved MR services due to late gestational age. However, logistical reasons including safety and cost were also barriers which reportedly delayed participants’ ability to obtain services before the authorized gestational limit. One said: ‘…No, [it’s] not so easy [to get MR]… Besides it’s costly, many are not able to afford it’ (R2, carried to term). For participants living in villages outside the city, physical access to providers was a commonly cited barrier. Most villages lack providers, but even when one is present, participants often are unaware of the services offered or how to access them. This participant described: ‘It is not easy to get these services from villages like ours, because there are no providers in the village. Most of the village women do not know where to go or whom to go. Cost is also a factor’ (S2, induced abortion). Though it is unclear, participants’ references to cost as a reason for delay may pertain to external costs outside of the procedure, such as transport to a clinic or opportunity cost of missing time at work, as well as the cost of services at private clinics.

### Knowledge of MR services

Participants were uninformed about their right to access safe MR services within the sanctioned period. None of the participants were aware of the gestational age window in which MR is permitted, even though about one-third of the participants reported knowing someone who had undergone MR. Regarding the various options for seeking MR, participants demonstrated a range of knowledge. Some said they were completely unaware of MR and where it was provided. Other respondents had heard of women receiving the service, but did not know how or where they had obtained it. Most participants were unaware of alternative methods for self-induction and reported that they did not seek out alternatives or try to self-induce. One said this was because of health and cost reasons:

*I did not even try to get any information or advice on this further. I did not try because I was scared to do so and also I had no money to go elsewhere. My mother brought abortion medicine from the pharmacy but I did not try it because I have heard that in later pregnancy it may damage my health. (M4, carried to term)*

Others were aware of clinic- or hospital-based options as well as medicines available at pharmacies, but did not mention methods of self-induction. For example: ‘I have heard that there are government and private hospitals that provide MR services. There are medicines in the pharmacies for MR. I don’t know any other options’ (D1, carried to term). Five women said that they had heard medication for termination was available at pharmacies and eight women said they had heard of traditional Ayurveda methods for abortion.

Overall, to the extent that participants were aware of MR and abortion services, they seemed to have learned the information by word of mouth or personal experience – not from any formal government, education or health care sources.

### MR experiences and quality of care

Of the eight participants who succeeded in obtaining abortion after initially being denied MR, there was substantial variation in the method of abortion they received and the overall quality of their experiences. Many described going to a clinic setting where they were given “medicine” and then had an abortion. It is not always clear what method was used in these cases: at least three participants reported taking pills (R8, R10, S2), one participant reported that she had been given oral contraceptive pills that induced abortion (R3, induced abortion), and the remaining four participants reported having a surgical procedure done (M3,R5,R9,S4). One participant described her experience: ‘A gynecologist did the MR. At the clinic they gave me some medicine to take. After that they performed MR at their operation theatre. It looked similar to other clinics. Neat and clean. Doctors provided consultation. I don’t know whether it was government approved or not. There were many patients and doctors in the clinic’ (R9, induced abortion).

Two of the eight participants who obtained abortions experienced severe bleeding. All three sought care and ultimately recovered. One woman recalled:

*I had excessive post abortion bleeding […] and had to be admitted to the hospital. They gave me three bags of blood. I had to go through D&C there. I had only wanted to get rid of the pregnancy. The clinic people told me not to tell anybody about the abortion service that I obtained here from them (R8, induced abortion).*

Another woman explained that she went back to the clinic after complications: ‘I was very scared thinking about what will happen to me. He advised me to visit again. He also advised me to push a saline. I had severe bleeding. Because too weak. Again it took medicine worth 250 taka. The same doctor gave me medicine again and I got cured’ (R10, induced abortion).

Most of the participants who obtained services reported that they were satisfied by the quality of care they received. Clinic environment and staff behavior were the two recurring factors that determined whether or not the quality of care was deemed adequate. One woman explained:

*The environment of the clinic was good, neat and clean. Privacy was also maintained. … I was fully satisfied with the service there. […] The doctor performed MR perfectly. She advised me to take a family planning method, gave me some medicine, advised me to take plenty of rest, avoid heavy work, and visit a doctor in case of any problem (S2, induced abortion).*

Participants’ perceptions of quality of care did not noticeably vary by facility type or provider type. One reported that the clinic’s authorized ability to provide MR services was irrelevant to the quality of care she received: ‘I didn’t feel anything. I felt good. The behavior of the provider was good. The place was neat and clean and the service was good. I do not know whether it was an approved clinic or not’ (S4, induced abortion). Providers included clinicians working in public and private clinics, retired government physicians, trained birth attendants and village doctors, and private physicians working out of their homes.

However, it was sometimes difficult to determine whether it was really the quality of care that the participants appreciated, or whether they were simply relieved to be done with the procedure and to not have experienced complications. For example, one participant said: ‘I am satisfied with the service. I had no complications. I have managed to escape from a big problem’ (R5, induced abortion). Only one of the two participants that experienced post-abortion complications expressed regret about the process: ‘If someone wants to go for MR like in my case, I would advise them to continue the pregnancy. I don’t want anybody to have the same experience like me. I have lost money and health too’ (R8, induced abortion). The other participant reported satisfaction with the procedure because it eventually achieved the desired outcome of ending the pregnancy.

## Discussion

Many women in Bangladesh do not receive desired MR services when they seek care at government sanctioned MR facilities. This study aims to understand the experiences of women who are denied MR in Bangladesh – specifically the decision-making process in seeking MR, reasons for denial of services, and women’s actions upon being turned away. The participants in this study were denied MR services because they presented past the government sanctioned gestational age limit, yet not a single participant was aware of the gestational limit for government approved MR, or that MR was regulated at all.

Of the 20 study participants, 12 sought a subsequent abortion elsewhere after being denied government sanctioned MR services. Of these 12 participants, eight were successful in obtaining an abortion. However, these subsequent abortions came with a high health risk: one-fourth of these participants experienced complications. Whether such high incidence of complications was due to lack of provider training in later abortion, the illicit nature of the services, or other factors is unknown. According to accounts from the eight participants who obtained an abortion, five likely had surgical procedures and three likely obtained medication abortions. Those estimated to have received surgical procedures used words like “performed” and “operation theatre” to describe their experiences, while those who likely received medication abortion mentioned “pills” or a delay between the interaction with the provider and the termination. Two of the participants who received medication abortions (beyond 12 weeks gestation) reported complications (R8, R10) and the third participant who received medication abortion said she had no complications but did report bleeding for 7-8 days (R3). Within our sample, participants sought abortion care at a range of private and public clinics, pharmacies, and from traditional providers. Given low levels of knowledge about government approved MR services among study participants, participant responses do not reveal whether the facilities they went to were authorized by the government to provide MR.

Participants in this study had a higher average education level compared to the national average for women in Bangladesh. Therefore, our results likely underestimate the rate at which women are denied MR services due to gestational age limitations, because the study participants may be better informed about the MR law and less likely to seek services beyond the limit. Further, our results may also underestimate the rate of illegal abortion attempts post denial and overestimate the percentage of participants who were able to successfully and legally terminate their pregnancies after denial.

As anticipated with an exploratory qualitative study, these findings are not generalizable or representative of all women in Bangladesh. Our results do not include the experiences of women who are under the age of 18 years or who seek abortion outside facility-based care, arguably the most vulnerable of women. In fact, the study team in Bangladesh followed a 14-year-old girl, not included in the sample discussed above but allowed under BAPSA Institutional Review Board (IRB) approvals because the girl was emancipated due to her married status. This girl terminated her pregnancy at a private clinic after being denied abortion due to advanced gestational age and experienced significant bleeding after her abortion. It is possible that the 11 women lost to follow up may differ from the study participants, according to the actions taken regarding their pregnancies; for example, they may have been more likely to attempt informal sector abortion, experience serious complications, or have been isolated or afraid to talk about their experiences for fear of legal repercussions. We also expect that the answers from women to questions about reaction to denial would have been more negative had participants been interviewed immediately after they were denied services rather than two months later. The advantage to conducting interviews after two months is that it gave us the opportunity to ask about participants’ experiences and subsequent courses of action following denial.

This is the first study to follow women denied MR services prospectively to investigate their experiences. Through in-depth interviews with respondents, we are able to begin to understand the challenges women face and their decision making around whether to continue to seek abortion services after denial. Our findings suggest a need to raise awareness about women’s rights to safe MR services in Bangladesh and to provide information on where, how and when women can access these services. To increase availability of MR for women and to reduce geographic barriers to care cited by numerous respondents, additional providers must be trained and approved. Finally, improved campaigns to educate women about contraception and pregnancy risk throughout the reproductive lifespan might prevent many unintended pregnancies.

## Conclusions

Overall, results from this study provide new insight into women’s experiences seeking abortion in Bangladesh: from factors associated with later presentation for MR, actions upon denial of services, safety of abortion procedures, and women’s awareness of and ability to exercise their rights to terminate a pregnancy. The future collection of large scale representative data will allow the investigation of many of the issues raised here. The full impact of receiving or being denied wanted MR services cannot be known without also following the women who were denied MR and carried the pregnancy to term. The health risks of childbirth have been shown to be significantly greater than terminating a pregnancy [2, 26], at least in high-resource settings such as the United States. It is plausible that this difference in risk would apply to Bangladesh as well, particularly within a developing country context where risk of maternal mortality/morbidity is relatively high in childbirth [14], but we do not have data on specific outcomes for the women in this study. Further, we do not know whether women’s concerns about affording another child or being able to care for existing children after having another child were borne out. To understand the total impact of denial of MR in Bangladesh, we would need to capture the consequences of receiving or being denied an MR procedure, of receiving an illegal abortion, and of carrying an unwanted pregnancy to term. These results can provide more targeted planning and programmatic data to inform interventions to improve the health and safety of Bangladeshi women faced with an unintended pregnancy.

## Abbreviations

BAPSA, Association for Prevention of Septic Abortion, Bangladesh; FWV, Family Welfare Volunteer; IRB, Institutional Review Board; LMP, last menstrual period; MR, menstrual regulation; MVA, manual vacuum aspiration; NGO, Non-Governmental Organization; SD, standard deviation

## Additional file

Additional file 1: Interview Guide. (PDF 335kb)
