# Supplementary material for: ‘How shall we survive’: a qualitative study of women’s experiences following denial of menstrual regulation (MR) services in Bangladesh
Source: Reprod Health. 2016 Jul 22;13:86. doi: 10.1186/s12978-016-0199-8 (PMC4957356; doi:10.1186/s12978-016-0199-8)
Supplement: Additional file 1: — Interview Guide. (PDF 335kb) [file 12978_2016_199_MOESM1_ESM.pdf]

Global Turn Away Study

UCSF/BAPSA

2013

Guideline for In-depth interviews with women

Introduction

- Introduce yourself and thank the participant for agreeing to meet you.
- Explain the purpose of the study and how the interview will be conducted (why s/he was selected, recording, what the information will be used for).
- Before you start the interview, obtain written informed consent.

Key reminders for Interviewer

- The participant was selected because she was denied to receive service from the place she first visited.
- The aim of the interview is to better understand:
  - Experiences seeking abortion services at various places.
  - Decision making process to end the pregnancy and choice of places
  - Impact on personal life and family wellbeing and future plan.
- The interview guide is only a guide. Try to cover all of the issues but there is considerable flexibility for the participant to tell you what s/he feels is important, and for you to find out about other interesting/related issues.
- Each question is followed by possible probes and issues to cover. You should only use these as examples or to generate ideas, not as a checklist.
- The ordering of the questions is intentional but you don't have to follow it if the respondent begins discussing something of interest which hasn't yet been covered; just return to the earlier question afterwards.
- Try to build trust and rapport with the respondent throughout the interview.
- Probe where necessary and clarify vague or contradictory information. Use prompts like:
  - Can you tell me more about that?
  - What do you mean exactly?
  - Am I right in thinking that…?
  - What do you think about…?
  - Do you mean that…?
  - Earlier you told me…but here you seem to be saying…Can you clarify this?

Informed Consent Form for In-depth interviews with women who were denied menstrual regulation services

<u>Informed Consent</u>

Hello! My name is……….......................…… I am from Association for Prevention of Septic Abortion, Bangladesh(BAPSA), a research organization based in Dhaka. We are involved in doing research on safe MR services with University of California San Francisco, USA. You might be knowing that MR –officially recognized as an interim method of establishing nonpregnancy-has been available in the government's family planning program as a public health measure since 1979. MR is included within the family planning program not as a contraceptive method, but rather as a backup for ineffective use of contraceptives, as no contraceptive is completely successful in preventing unwanted pregnancy. MR is widely available in Bangladesh and the government has allowed providing services through all Medical College Hospitals, District hospitals, Mother and Child Welfare Centers, Upazila Health Complexes, specialized centers, Union Health and Family Welfare Centers and large number of NGO clinics to provide safe MR services to women.

Purpose of the study

The purpose of this study is to explore women's experiences after being denied a wanted MR.

Procedure

To explore these questions, we invite you to take part in this study. If you accept, I will ask you a series of questions. The questions pertain to your experiences seeking an MR, your knowledge about the abortion law and services, reasons for being denied an MR and experiences with MR services generally. You are being invited to participate in this study because I feel that the information you would provide can contribute much to our study.

If you do not wish to answer any of the questions posed during the interview, you may say so and I shall move on to the next question. The interview will take place in this room and there will be no one else present in this room. The information recorded is considered confidential, and no one else except our study team will have access to the information documented during the interview.

The expected duration of the interview is about an hour.

Risks and Discomforts

You may feel uncomfortable talking about some topics. However, I do not wish this to happen, and you may refuse to answer any question or not take part in a portion of the interview if you feel the questions are personal or if talking or writing about them makes you uncomfortable.

Benefits

There will be no direct benefit to you, but your participation is likely to help us to find more about the reasons for not receiving abortion/MR service and it impact on women's lives.

Confidentiality

All information provided by you will be strictly confidential and will only be used for analysis in the study. The information provided will not be shown to other persons. I will write down information from the questions you answer but your name will be kept secret. To protect your privacy, your names will not be written in any of these pages. This information will be kept in a separate locked cabinet and once the study is completed, this information will be destroyed.

Right to refuse or withdraw

You do not have to take part in this research if you do not wish to do so and refusing to participate will not affect your future access to services. You will still have all the benefits that you would otherwise have at this health facility.

You may stop participating in the interview at any time that you wish. This will not affect your right to access health services including abortion services from this or any health facilities.

Who to contact

If you have any questions you may ask those now or later. If you wish to ask questions later, you may contact Altaf Hossain, Director BAPSA.

This study has been reviewed and approved by the Bangladesh Medical Research Council (BMRC),which is a national committee whose task is to make sure that research participants are protected from harm. If you wish to find out more about BMRC please contact Dr.---------------, Phone no.

I have read the foregoing information, or it has been read to me. I have had the opportunity to ask questions about it and any questions that I have asked have been answered to my satisfaction. I consent voluntarily to participate as a subject in this study and understand that I have the right to withdraw from the study at any time without in any way affecting my further access to services.

____

Date Signature or thumb print of Participant

____

Date Signature of Interviewer

01. Participant ID: ☐☐

02. Division: ____________________________ **04 . District:** ________________________

03. Upazilla: ____________________________ **05. Union:** __________________________

06. Facility name (Where the respondent rejected): _____________________________________

07. Interviewer Name:_____________________________ **08. Date of Interview:** ___________

09. Time started: __________________________ **10. Time End:**________________________

11. Supervisor’s Signature: __________________________ **12. Date:**_____________________

1. Participant Background

Topic Focus	Core questions	Additional questions or prompts	
**Personal Background**	• Could you please tell me about yourself	1.1. Present Age: ☐☐	1.2. Age at marriage: ☐☐
		1.2. Number of living Children: ☐☐ Son/s: ☐☐ Daughter/s	
		1.4. Ethnicity:___________	
		1.5. Place of living : ☐ City ☐ Town ☐ Village	
		**1.6. Socio- economic status of Family:**	
		1.6.1. Own land:	☐☐ Acres ☐☐ Decimals
		1.6.2. Monthly Income:	BDT-☐☐☐☐☐☐☐
		1.6.3. Material of your house:(it is building or Kaccha or Mud house or Corrugated iron sheet)	___________
		1.6.4. Main occupation of the respondent	___________
		1.6.5. Main occupation of the spouse:	___________
		1.7. Education of the Respondent:	☐No education<br>☐Primary (1st-5th)<br>☐Secondary (6th -10th)<br>☐College or Higher (11th+)<br>☐Don't Know/Refused
		1.8. Education of the Spouse:	☐No education<br>☐Primary (1st-5th)<br>☐Secondary (6th -10th)<br>☐College or Higher (11th+)<br>☐Don't Know/Refused
		1.9. Currently living with partner or not.	☐ Living with partner<br>☐ Living elsewhere

2. Decision making process

**Decision making**	2.1. I would like you to tell me what was happening in your life at the time of this pregnancy?	2.1.1. When did you first discover that you were pregnant? Who did feel about that time?<br><br>2.1.2 What was your partner's initial reaction to this pregnancy?
	2.2 Why was the pregnancy considered unintended?	2.2.1. Was it due to method failure?/ Was it related to forced sex?/ Was it due to lack of availability of contraceptives?/ Was it due to improper use of methods?/ Or any other reasons, please tell that.<br><br>.

	2.3 Do you recall the circumstances that urged you to think in that direction (advice for abortion)?	2.3.1. What circumstances initially compelled you to decide to go for MR (terminate the pregnancy)?<br><br>2.3.2 Do you remember talking to anyone before discussing the decision to go for an MR? If so, who did you talk to about the pregnancy?<br><br>2.3.3 What advice were you looking for? What information did you receive?<br><br>2.3.4 Was the decision to have MR (abort) influenced by the advice given by others you may have spoken with? If so, who influenced your decision?<br><br>2.3.5 Was there anyone else involved in this discussion for having an MR (about terminating the pregnancy)?

		2.3.6 Who had the final say on the decision?<br><br>2.3.7 What considerations did you have to think about in making this decision?<br><br>2.3.8 Were there any difficulties (impediments) that you faced in making this decision?<br><br>2.3.9 How long did it take from the initial decision making to actually visiting a provider?<br><br>2.3.10 What factors do you think kept you from coming to the clinic earlier in your pregnancy?

3. Abortion seeking behavior

**Experiences with the clinic visit**	3.1 Could you please tell me what happened at the clinic/ hospital when you went for an abortion	3.1.1 How did you feel about what happened?<br><br>3.1.2 What were some of the reasons you weren't able to get an MR (abortion) at [clinic name] that day?<br><br>3.1.3 Did you have an ultrasound test before coming to this facility?
**Subsequent attempt for abortion**	3.2 What did you do after you left the clinic?<br><br>Did you ever consider getting an abortion elsewhere/going to another clinic/provider?<br><br>If yes, can you tell me about this? *(please ask about all the process and all places visited)*	3.2.1 Who do you talk to?<br><br>3.2.2 Who, if anyone, did you seek advice from about what to do next?

	Why did you or did you not seek an abortion elsewhere?	3.2.3 Do you remember talking to anyone? From where? From whom? What information advice you were given?
	3.3 <u>If sought MR/abortion elsewhere</u><br><br>Tell me what happened when you went to this provider. [*try to capture the chronology of events, concerns or dilemmas that were occurring, and what emotions were involved]*<br><br>Was the abortion successful? If no, why not?	3.3.1 Without giving me anyone's name, what kind of person did you seek an MR/abortion from/where did you go?<br><br>3.3.2 What happened?<br><br>3.3.3 How did you feel about what happened? (*probe to see if she felt it was a safe place/if she felt safe, probe to see if she is aware about government approved place*)<br><br>3.3.4 How much did the MR/ abortion cost?

		3.3.5 Were you satisfied with the outcome? What concerns do you remember having? Did you get any advice about seeking medical care after the MR/ abortion? What was the advice?<br><br>3.3.6 Did you experience any complications following the MR/abortion?<br>If so, what kind of complications did you face?<br><br>3.3.7 Did you seek medical or other care after the MR/ abortion? What medical care did you seek and why?<br><br>3.3.8 Is there anything you wish that you had known before going to this provider?

**Attempted self-induction for abortion**	***3.4. If not visited elsewhere for an abortion:***<br><br>Did you ever consider ending the pregnancy yourself?<br><br>If yes, can you tell me about that thought process?<br><br>Did you try to end the pregnancy yourself? Why or why not?<br><br>**<u>If tried by herself:</u>**<br>How did you try to end the pregnancy yourself?<br><br>**<u>If used medicines (both effective or ineffective) :</u>**<br><br>Can you tell me about your experience trying to end the pregnancy yourself?	3.4.1 Did you seek any information or advice about it?<br><br>3.4.2 From where? From whom? What information advice you were given?(*ask separately for each source of advice*)<br><br>3.4.5 What happened?<br><br>3.4.6 How did you feel about what happened?<br><br>3.4.7 Without giving me anyone's name, where did you get the drugs?<br><br>3.4.8 How much it cost?<br><br>3.4.9 What, if any, directions did you get about using the drugs?

	Was the abortion successful?<br><br>**<u>If used other traditional methods:</u>**<br><br>Can you tell me about your experience trying to end the pregnancy yourself?<br>Was the abortion successful?	3.4.10 Without giving me anyone's name, where did you get directions about using the drug?<br><br>3.4.11 Did you experience any complications following the MR/abortion?<br>If so, what kind of complications did you face?<br><br>3.4.12 Did you seek medical or other care after the MR/abortion?<br><br>3.4.13 What medical care did you seek and why?<br><br>3.4.14 What, if any, advice or information did you get about ending the pregnancy yourself?<br><br>3.4.15 What happened?<br><br>3.4.16 How did you feel about what happened?<br><br>3.4.17 Did you experience any complications following the MR/abortion?<br>If so, what kind of complications did you face?

		3.4.18 Did you seek medical or other care after the MR/ abortion? What medical care did you seek and why?

4. Future plan and impact on family life and wellbeing

**Impact**	**4.1Ask if woman is still pregnant**<br><br>What are your plans for after the baby is born?	4.1.1 How has your relationship with your family changed now that you are having a baby?<br><br>4.1.2 How has your relationship with your partner changed now that you are having a baby?<br><br>4.1.3 How do you think that having a baby will affect your life?

5. Knowledge about the laws and services

**Knowledge about the law and services**	5.1 Tell me what you know about the abortion law in Bangladesh?<br><br>Do you remember when you and your partner decided to terminate the pregnancy, were either of you aware about the abortion law in our country?	5.1.1 Have you heard anything about the new abortion law?On what conditions a woman can have legal abortion in Bangladesh? *(Be sure to note carefully any misinformation or incorrect information respondent might have about the existing law)*
	5.2 Of the women you know, how many might have terminated a pregnancy at some time or the other? *(Probe: Think of your friends, relatives, and neighbors)*	5.2.1 Without telling me their names, how do you know they may have terminated a pregnancy? How well do you know them?
	5.3 Around here, if a woman could not have a menstrual regulation and wanted to terminate a pregnancy, do you think it would be easy or difficult for her to do it? Why?	Reasons/ Barriers:

	5.4 If a woman could not have a menstrual regulation, can you tell me what are some ways that people say a woman could terminate a pregnancy?	Note:
	5.6 If a woman could not have a menstrual regulation, are there places or people a woman could go if she wanted to terminate a pregnancy? Where? To whom?	Note:
**Advice for others seeking abortion**	5.7 If someone approached you about an unintended pregnancy, some one who is experiencing the same situation that you had and they asked you what they should do, what would you say to them? Why?	Note:
